# Genome Sequence of *Anoxybacillus flavithermus* Strain AK1, a Thermophile Isolated from a Hot Spring in Saudi Arabia

**DOI:** 10.1128/genomeA.00604-15

**Published:** 2015-06-04

**Authors:** Amjad Khalil, Neelamegam Sivakumar, Sami Qarawi

**Affiliations:** a Department of Biology, King Fahd University of Petroleum and Minerals, Dhahran, Saudi Arabia; b Biosciences Core Lab, King Abdullah University of Science and Technology, Thuwal, Jeddah, Saudi Arabia

## Abstract

*Anoxybacillus flavithermus* strain AK1 was isolated from Al-Ain Alhara, a thermal hot spring located 50 km southeast of the city of Gazan, Saudi Arabia (16°56ʹN, 43°15ʹE). The sequenced and annotated genome is 2,630,664 bp and encodes 2,799 genes.

## GENOME ANNOUNCEMENT

The thermophile *Anoxybacillus flavothermus* was first discovered in a hot spring by Heinen et al. (1). The strain was initially recognized as *Bacillus flavothermus* based on the proteins it was found to express by biochemical analyses (2–4). The genus name of the strain remained unchanged until 2000, when Pikuta and coworkers (5) conducted a detailed study primarily based on the genetics of a similar strain, *A. pushchinensis* K1^T^, isolated from animal manure. The phenotypic features and DNA–DNA hybridization analyses revealed that the strain was a facultative anaerobe, making it different from other members of the genus *Bacillus*. Furthermore, *A. pushchinensis* K1^T^ and *B. flavothermus* were phylogenetically clustered together and, consequently, reclassified as *Anoxybacillus flavothermus*. This lead to the introduction of the new genera *Anoxybacillus*, whose members are similar to the members of the group bacillus but can survive without oxygen (5).

*Anoxybacillus flavithermus* strain AK1 was isolated from Al-Ain Alhara, a thermal hot spring located 50 km southeast of the city of Gazan, Saudi Arabia (16°56ʹN, 43°15ʹE).

*A. flavithermus* strain AK1 was grown in TT medium at 55°C for 24 to 48 h (ATCC medium 697). Then, cells were harvested by centrifugation of 10-mL aliquots of medium, and the bacterial pellets were spread over TT agar medium in a benchtop incubator. A number of distinctive colonies were picked and transferred to fresh TT agar plates thrice to obtain a pure culture, which was then further incubated at 55°C. The total bacterial DNA was extracted from all isolates using the Norgen Biotek bacterial genomic DNA isolation kit following the manufacturer’s guidelines. Lastly, the complete genome of *A. flavithermus* AK1 was sequenced using a 454 genome sequencer (FLX Titanium; Roche).

Whole-genome shotgun assembly was performed using Newbler version 2.5.3. Contigs shorter than 1,000 bp were deleted. A total of 50 contigs produced a genome sequence of 2,630,664 bp in length with a GC content of 42.67%. The complete genome sequence comprised 2,799 predicted genes, among which 2,724 (97.32%) were protein-coding genes and 75 were RNA genes (2.68%). Furthermore, 18 rRNA genes (six 5S rRNA, seven 16S rRNA, and five 23S rRNA genes), and 57 predicted tRNA genes were recognized in the genome. Finally, a total of 2,413 genes (86.21%) were assigned a putative function, and the remaining genes were annotated as hypothetical proteins.

Phylogenetic analysis was performed using 16S rRNA sequences with the BioNJ algorithm implemented in SeaView version 4.5.3, and 16S rRNA sequences of *Anoxybacillus*-type strains were found using GenBank (http://www.ncbi.nlm.nih.gov/nucleotide) database. According to phylogenetic analysis, *Anoxybacillus* strain AK1 is most closely related to *Anoxybacillus flavithermus*. The phylogenetic position of *A. flavithermus* AK1 indicates that the strain is most closely related to *Anoxybacillus flavithermus* AE3, *Anoxybacillus* sp. SW, and *Anoxybacillus* sp. DT3-3.

### Nucleotide sequence accession numbers. 

The draft genome sequence for *A. flavithermus* AK1 has been deposited in GenBank under the accession number APCD00000000. The 50 contigs have been deposited under the accession numbers APCD01000001 to APCD01000050.
